# Observations on the Case of a Hernia, Where Strangulation Was Produced by Inflammation, and Which Continued after the Operation

**Published:** 1803-09-01

**Authors:** M. Renoult

**Affiliations:** first Surgeon in the Army


### Observations *on the* Case *of a* Hernia, *where* Strangulation *was produced by* Inflammation, *and which continued after the* Operation; *by* M. Renoult, *first Surgeon in the Army.*

A Young officer had a hernia, which he retained in place for a considerable time by means of a bandage. While encamped in the Low Countries, he was thrown from his horse, after a long continued restiveness and leaping, which forced the intestine out, attended with much pain. The patient made immediate and fruitless efforts to reduce it, and was brought back to his hut, where many trials were made unsuccessfully with the same intent, during ten hours; after which he was sent to the military hospital, under the direction of the author. To the means already employed, the warm bath was added, but the great sensibility of the tumour precluded the possibility of recurring to the operation of the taxis. As the patient got hourly worse, the operation appeared to M. R. the only means by which relief could be expected, and which was performed six hours after his entrance into the hospital, and sixteen from his accident. The intestine, which alone formed the hernia, was of a deep red colour, and had adhered but in a trifling degree to the surrounding parts. The portion of the intestine was considerable, and the omentum seemed to have no share whatever in the formation of the tumour; after disengaging the ring, and finding finding no contra-indication to the reduction of the intestine, the operator gently drew out a larger portion of the intestine, with a view of facilitating the reduction of the protruded part, which was considerably distended. On the most trifling effort the intestine slipped into the abdomen. The operator counted on the disappearance of the symptoms, and the patient did experience some relief for a few hours, after which, hiccup and vomiting came on; the belly was tense, the pulse feeble, and the patient died eleven hours after the operation. Considering death to have been produced by gangrene of the intestine, the author wished to assure himself by examining the body. He found the omentum rolled on itself, and embracing tightly the ileum, and which formed a noose, both extremities of which adhered strongly to the mesentery and the middle of the intestine, so that the continuity of the intestinal tube was interrupted by this kind of ligature. The author thinks it probable that, originally, the omentum formed in part, perhaps wholly, the hernia, and that this viscus, long dragged down, became lengthened, and floated in the abdomen. He conceives it equally probable, that in the fall which the deceased experienced, the intestine first protruded, closing completely the ring, so that the omentum, which at this moment was found to tie it in some measure, was only engaged in its curvature to a small degree, and consequently did not a little contribute to its strangulation. Hence the rapid adhesions which were formed between them, and which escaped into the abdomen as soon as the opposition at the ring was removed; but that which was produced by the omentum always exerting, perhaps too increased by the position of the parts after reduction, kept up the symptoms, and caused the fatal termination. The author tells us, that if he had been aware of this state of the parts he would have prevented their return till he had freed the intestine from its adhesions; but the omentum was not nor could not be perceived; the appearance of the intestine was favourable to its return, and there was no reason whatever for suspecting the state of the parts within the ring. This case deserves attention, in as far as it adds to the instances, not very numerous, of strangulation produced by the omentum or mesentery within the ring, and which shews the necessity for the most scrupulous examination into the state of the parts before their return.

*June* 10, 1803.

CASE

